# Effects of puerarin on the Akt signaling pathway in bovine preadipocyte differentiation

**DOI:** 10.5713/ajas.19.0004

**Published:** 2019-05-28

**Authors:** Jinyan Yun, Yongsheng Yu, Guoli Zhou, Xiaotong Luo, Haiguo Jin, Yumin Zhao, Yang Cao

**Affiliations:** 1 Branch of Animal Husbandry, Jilin Academy of Agricultural Sciences, Changchun 130033, China; 2 Key Laboratory of Beef Cattle Genetics and Breeding in Ministry of Agriculture and Rural Agriculture, Changchun 130033, China; 3 College of Life Science, Liaocheng University, Liaocheng 252000, China

**Keywords:** Puerarin, Adipogenesis, Akt Signaling Pathway, Preadipocyte Differentiation, Bovine

## Abstract

**Objective:**

Puerarin has the potential of regulating the differentiation of preadipocytes, but its mechanism of action has not yet been elucidated. Adipocytes found in adipose tissue, the main endocrine organ, are the main sites of lipid deposition, and are widely used as a cell model in the study of *in vitro* fat deposition. This study aimed to investigate the effects of puerarin on adipogenesis *in vitro*.

**Methods:**

Puerarin was added to the culture medium during the process of adipogenesis. The proliferation and differentiation of bovine preadipocytes was measured through cell viability and staining with oil red O. The content of triacylglycerol was measured using a triglyceride assay kit. The mRNA and protein expression levels of adipogenic genes, peroxisome proliferator-activated receptor-γ (PPARγ) and CCAAT/enhancer-binding protein-α, were measured using quantitative real-time polymerase chain reaction and western blotting, respectively.

**Results:**

The addition of puerarin significantly increased adipogenesis of bovine preadipocytes and enhanced the mRNA and protein level expression of PPARγ (p<0.01). The expression of P-Akt increased after adipogenic hormonal induction, whereas puerarin significantly increased PPARγ expression by promoting the Akt signaling component, P-Akt. The mechanism of adipogenesis was found to be related to the phosphorylation level of Ser473, which may activate the downstream signaling of the Akt pathway.

**Conclusion:**

Puerarin was able to promote the differentiation of preadipocytes and improve fat deposition in cattle. The mechanism of adipogenesis was found to be related to the phosphorylation level of Ser473.

## INTRODUCTION

Yan Yellow cattle, one of the breeding varieties of cattle in China, have become increasingly popular due to their excellent meat quality. The fleshy traits of Yan Yellow cattle are comparable to that of Wagyu and Hanwoo cattle. The fat content of beef is significantly associated with several meat quality traits. The deposition of intramuscular fat is a dynamic balanced process that involves the anabolism and catabolism of lipids, including processes such as fat synthesis, fat degradation, and fatty acid transportation. When anabolic ability is greater than catabolic ability, fat deposition increases. Conversely, when catabolic ability is greater than anabolic ability, the original balance is destroyed, and fat deposition decreases.

Adipose tissue is a loose connective tissue that consists of adipocytes and functions as the main organ of energy storage and endocrine function in animals. Adipose tissue not only plays an important role in regulating metabolism, but also regulates the amount of fat deposition, which has an impact on animal health and meat quality [1–6]. Adipogenesis includes preadipocyte proliferation and differentiation into mature adipocytes, which is regulated by various cytokines and signaling pathways. When the energy balance has not been disrupted, the size and number of adipocytes have no effect on total adiposity [7]. Therefore, regulation of biological processes, including the formation, cellular proliferation, differentiation and apoptosis of adipocytes are very important for fat deposition control.

An increase in the number of adipocytes caused by cell proliferation and the increase in volume of adipocytes caused by adipogenesis are the main causes of fat deposition *in vivo* [8]. For beef cattle, fat cells proliferate during all life stages. The progenitor cells of adipocytes gradually decrease along with the increase in age, which is also the cause of decrease of new adipocytes [9–11].

Puerarin, a major isoflavone glycoside found in Kudzu root (*Pueraria lobata*), is the main monomer component of crude isoflavones in *Pueraria lobata* extract. Previous studies have reported that Puerarin treatment can prevent diet induced obesity and glucose tolerance [12,13]. However, Lee indicated that Puerarin treatment significantly enhances the differentiation of 3T3-L1 preadipocytes and is accompanied by an increase in lipid accumulation. The controversial effect of puerarin on weight gain and food intake has prompted us to further investigate the effect of puerarin on adipogenesis in an *in vitro* model [14,15]. This study aims to investigate the molecular mechanism of puerarin on adipogenesis *in vitro*.

## MATERIALS AND METHODS

### Ethics statement

The protocol of this study was approved by the Animal Ethics Committee of Jilin Academy of Agricultural Sciences (AWEC 2017A01, 9 March 2017). The cattle were handled in accordance with good animal practice as required by the Animal Ethics Procedures and Guidelines of the People’s Republic of China.

### Materials

Puerarin (CAS:3681-99-0) (Beijing Century Aoke Biotechnology Co., Ltd., Beijing, China); Dexamethasone (DEX), 1-methyl-3-isobutylxanthine (IBMX), insulin (Sigma, St. Louis, MO, USA); dimethyl sulfoxide (DMSO), TRIzol Reagent (Ambition Scotland, UK), CellTiter 96 AQueous One Solution Cell Proliferation Assay (Promega Corporation, Madison, WI, USA).

### Cell culture and preadipocyte differentiation

The preadipocytes were extracted from dorsal subcutaneous adipose tissue of Yan Yellow cattle. Large blood vessels and fascia of adipose were removed, then, adipose tissues were cut into pieces of approximately 1 mm^3^. The tissue samples were digested with collagenase I, and then seeded into 75 mm^2^ plates at a density of 6×10^5^ cells/plate. Preadipocytes were maintained in Dulbecco’s modification of Eagle’s medium (DMEM) (CORNING, Manassas, VA, USA) containing 10% fetal bovine serum (Gibco BRL Life Technology, Grand Island, NY, USA) and 1% penicillin-streptomycin solution (100×) (BBI Life Science Corporation, Hong Kong, China). Two days after the cells reached confluence, the medium was removed, induced by treatment with differentiation medium I (10 μg/mL insulin, 0.5 mM IBMX and 1.0 μM DEX) for 48 h. The cells were supplemented with DMEM containing 10 μg/mL insulin (differentiation medium II) for an additional 48 h and further cultured in DMEM without insulin every two days until day 8. Puerarin was dissolved in DMSO and diluted to a concentration of 10 mM with DMEM, and then the puerarin stock solution was added to the differentiation medium.

### Preadipocyte proliferation

Absorbance was measured using a CellTiter 96 AQ_ueous_ One Solution Cell Proliferation Assay. Puerarin samples were titrated at a concentration of 5 mM in column 12, and serial twofold dilutions were performed across the plate in column 2 (to 5 μM). Column 1 was used as the negative control: Dulbecco’s modification of Eagle’s medium (and supplements) without puerarin. The cell suspension (5,000 cells) was dispense into all the wells of the plate prepared. The final concentration of Puerarin in the medium was adjusted to either 0, 2.5, 5, 10, 20, 40, or 80 μM. After the plate was incubated for 48 hours, 20 μL of CellTiter 96 AQueous One Solution Reagent was added and absorbance at 490 nm was recorded using a 96-well plate reader.

### Oil red O staining and lipid droplet accumulation in adipocytes

Lipid droplet accumulation was measured using oil red O staining. Adipocytes were washed twice with phosphate buffer saline (PBS) and fixed in 4% paraformaldehyde for 30 min at 37°C after the basal medium was removed. Then, the cells were washed twice again with H_2_O and stained using 0.5% oil red O stock solution in 6:4 (v/v) isopropyl alcohol: H_2_O for 30 min, at room temperature. The stained cells were washed three times at least and subsequently photographed using a Nikon ECLIPSE TS100/TS100-F microscope (Nikon Corporation, Tokyo, Japan). Oil red O was extracted with isopropyl alcohol and absorbance was measured at 510 nm using a microplate photometer (Thermo Fisher Scientific, Waltham, MA, USA).

### Quantification of triacylglycerol content

The adipocytes were washed twice with PBS and solubilized in a lysis buffer and incubated for 10 min at room temperature. The supernatants were collected and treated at 70°C for 10 min, followed by centrifugation at 2,000 rpm for 5 min. Then the working solution was mixed at 4:1 (v/v) R1:R2. Glycerin Standard was dissolved by doubling dilution, with the final concentration being 4,000, 2,000, 1,000, 500, 250, 125, or 62.5 μM. Absorbance of OD_550nm_ was measured using a Microplate photometer (Thermo Fisher Scientific, USA).

### RNA extraction and real-time quantitative polymerase chain reaction analysis

Total RNA was extracted using TRIzol reagent (Ambition Scotland, UK), according to the manufacturer’s instructions. The extracted RNA was reverse transcribed into cDNA using a GoScript Reverse Transcription System (Promega Corporation, USA) as described. Primer sequences of the target genes were as follows: peroxisome proliferator-activated receptor-γ (PPARγ) (5′-ATTATTCTCAGTGGAGACCGCC-3′ and 5′-CAAG- GCTTGCAGCAGATTGT-3′) CCAAT/enhancer-binding protein-α (C/EBPα) (5′-CAGAAGGGTTCT GCCGCTAT-3′ and 5′-CTGCTGGGCTTGTATCCACA-3′) β-actin (5′-AGATCAAGATCATC- GCGCCC-3′ and 5′-TA ACGCAGCTAACAGTCCGC-3′). The relative mRNA expression level was qualified using SYBR Green PCR Master Mix (Roche, Mannheim, Germany) and Roche Light Cycler 480 real-time fluorescent quantitative polymerase chain reaction (PCR) system. As for the relative gene expression normalized for internal control, β-actin was selected. The reaction mixtures were incubated for pre-denaturation at 95°C for 5 min, followed by 40 PCR cycles: 10 s at 95°C, 15 s at 60°C, and 20 s at 72°C. Each experiment was performed in triplicate.

### Western blotting

After 8 days of differentiation in the presence of puerarin, the adipocytes were lysed using RIPA lysis and extraction buffer (Thermo Fisher Technology, USA) containing protease and a phosphatase inhibitor cocktail (Thermo Fisher Technology, USA). Protein content was determined using a BCA protein assay reagent (Beyotime Biotechnology, Beijing, China). The polyvinylidene difluoride (PVDF) membranes (Millipore Corporation, Bedford, MA, USA) were incubated with the following antibodies: Akt antibody (dilution, 1:1,000; catalog no.,#9272; Cell Signaling Technology, Danvers, MA, USA), phospho-Akt (Ser473) antibody (dilution, 1:1,000; catalog no., #9271; CST), PPARγ (dilution, 1:1,000; catalog no., ab45036; Abcam, Cambridge, MA, USA) C/EBPα (dilution, 1:500; catalog no., abs123637a; Absin Bioscience Inc., Shanghai, China) and anti-β-actin (dilution, 1:1,000; catalog no., ab6276; Abcam) at 4°C overnight, followed by incubation with anti-mouse horseradish peroxidase (HRP)-conjugated secondary antibody (dilution, 1:1,000; catalog no., #7076; CST) or Anti-rabbit IgG HRP-linked antibody (dilution, 1:1,000; catalog no., #7074; CST) at room temperature for 1 hour. Immunoreactive proteins were detected using a Super ECL Plus system (Applygen Technology Inc., Shanghai, China) and quantified using Image J software.

### Statistical analysis

All data are expressed as mean±standard deviation. Statistical significance is defined when p values are less than 0.05 and a p value of <0.01 was considered to be extremely significant difference. One-way analysis of variance and post hoc test were used for all statistical analyses using GraphPad Prism software (GraphPad Prism software, Inc., La Jolla, CA, USA). Each value was obtained as the average of three independent experiments.

## RESULTS

### Puerarin enhances bovine adipocyte differentiation

**Figure 1:**
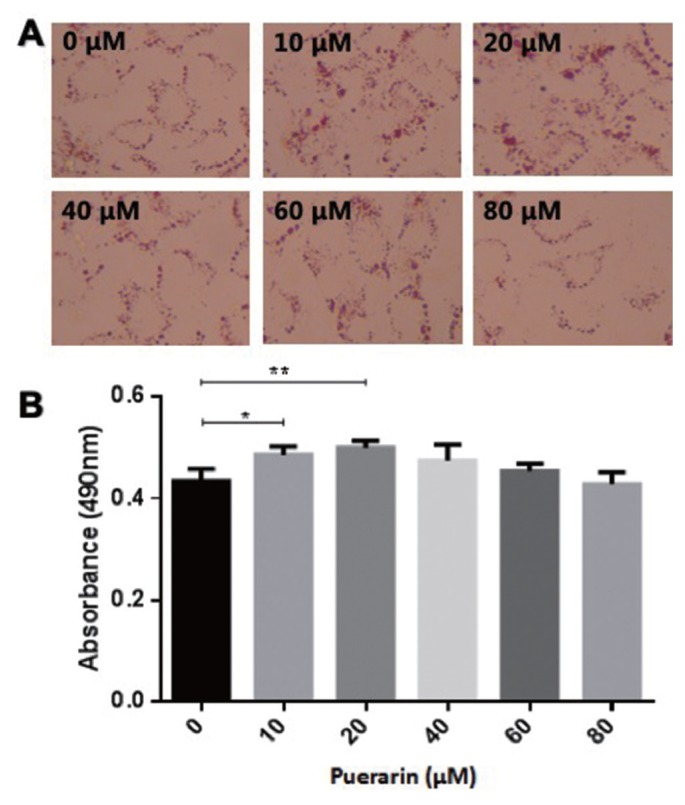
Effects of puerarin on intracellular lipid accumulation in preadipocytes of Yan Yellow cattle. Puerarin stimulated adipogenesis of primary preadipocytes. The effect of puerarin on the differentiation of cattle primary preadipocytes was assessed using oil red O stain, with all measurements being taken on day 8 of differentiation by induction of puerarin at the concentrations indicated. (A) Mature adipocytes stained with oil red O (400×). The final concentration of puerarin in the medium was adjusted to 0, 10, 20, 40, 60, or 80 μM. (B) Mature adipocytes stained with oil red O and lipid accumulation were quantified through extraction using isopropyl alcohol, as described under Materials and Methods. Data are presented as mean±standard deviation of experiments performed in triplicate; one-way analysis of variance. * p<0.05 compared with the control; ** p<0.01 compared with the control.

**Figure 2:**
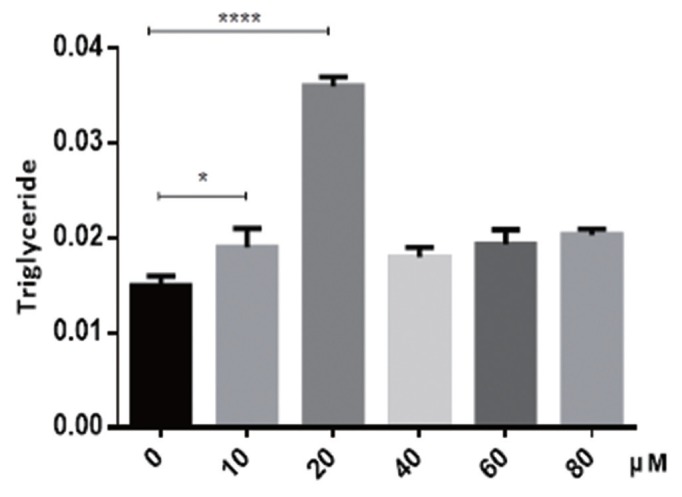
Effect of puerarin on triacylglycerol content of preadipocytes in Yan Yellow cattle. The supernatants of the adipocytic lysate were collected and detected using a Triglyceride assay kit at differentiation day 8. Triacylglycerol content was measured to quantify intracellular lipid content. Data are presented as mean±standard deviation of experiments performed in triplicate; one-way analysis of variance. * p<0.05 compared with the control; **** p<0.0001 compared with the control.

In order to determine the potential impact of puerarin on preadipocyte differentiation, preadipocytes were induced using the differentiation medium in the absence or presence of puerarin (0, 10, 20, 40, 60, or 80 μM). The accumulated lipid droplet was measured using oil red O at day 8 (Figure 1A). As shown in Figure 1B, doses of 10 and 20 μM of puerarin were observed to produce a significantly increased intracellular fat content compared to that of control cells, as measured using a plate reader at 490 nm. These results were also confirmed using a triacylglycerol (TG) content assay (Figure 2). Triglyceride content of 10 μM puerarin-treated preadipocytes were significantly higher (p<0.05), compared with that of the control group, while preadipocytes treated with 20 μM puerarin had an extremely significantly increased triglyceride content (p<0.0001). Puerarin (10 μM or 20 μM) enhanced adipocyte differentiation as evidenced by the increased triglyceride accumulation in adipocytes. Therefore, subsequent experiments further studied the effect of puerarin on preadipocyte differentiation mechanism using these two concentrations of puerarin.

### Puerarin regulates the expression of adipogenic transcription regulator C/EBP**α** and PPAR**γ**

**Figure 3:**
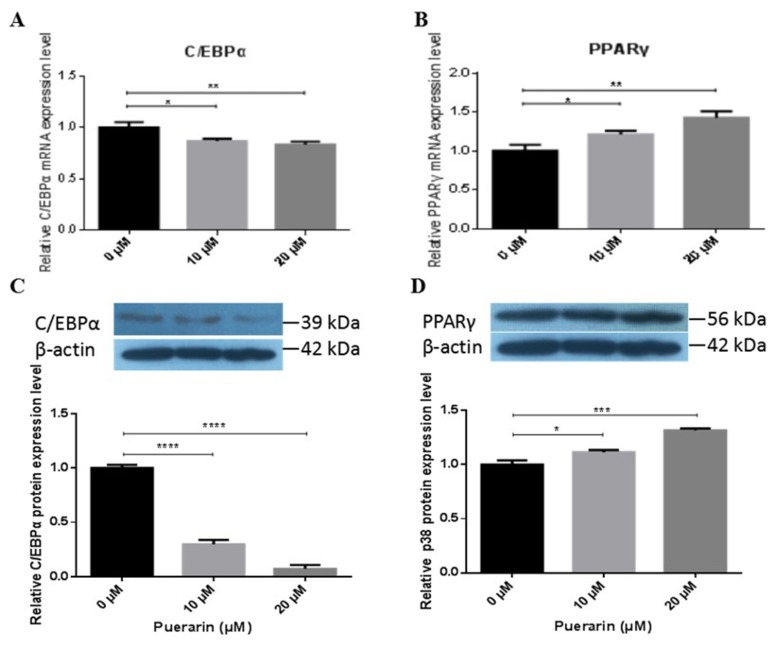
Effects of puerarin on the expression of adipogenesis-related transcription factors C/EBPα and PPARγ. (A), (B) The relative mRNA expression level was qualified using the SYBR Green PCR Master Mix and Roche Light Cycler 480 real-time fluorescent quantitative PCR system on differentiation day 8. The reaction mixtures were incubated for pre-denaturation at 95°C for 5 min, followed by 40 PCR cycles: 10 s at 95°C, 15 s for 60°C, and 20 s for 72°C. Each experiment was carried out in triplicate. The relative mRNA expression of C/EBPα and PPARγ were determined after the pre-adipocytes were incubated with the differentiation medium in the presence or absence of 10 μM or 20 μM puerarin for 8 days. (C), (D) After 8 days of differentiation in the presence of puerarin, the adipocytes were lysed, and western blotting was applied to the PVDF membranes. Immunoreactive proteins were detected using a Super ECL Plus system and were quantified using Image J software. The relative protein expression of C/EBPα and PPARγ were determined after the pre-adipocytes were incubated with the differentiation medium in the presence or absence of 10 μM or 20 μM puerarin for 8 days. Data are presented as mean±standard deviation of experiments performed in triplicate; one-way analysis of variance. C/EBPα, CCAAT/enhancer-binding protein-α; PPARγ, peroxisome proliferator-activated receptor-γ; PCR, polymerase chain reaction; PVDF, polyvinylidene difluoride. * p<0.05 compared with control; ** p<0.01 compared with the control; *** p<0.001 compared with the control; **** p<0.0001 compared with the control.

In order to identify the expression levels of C/EBPα and PPARγ, preadipocytes were cultured at the indicated concentrations (10 and 20 μM) of puerarin. As shown in Figure 3, consistent with its morphology, the mRNA expression level of C/EBPα decreased significantly in a dose dependent manner (p<0.05, 10 μM; p<0.01, 20 μM). The mRNA expression level of PPARγ had significantly improved in the same manner (p<0.05, 10 μM; p<0.01, 20 μM) (Figure 3A, 3B). The protein expression levels of C/EBPα and PPARγ obtained through western blot analysis showed similar patterns compared with that of the mRNA expression levels (Figure 3C, 3D). These results indicate that puerarin at concentrations of 10 μM and 20 μM significantly change C/EBPα and PPARγ expression, compared with the control group during adipocyte differentiation. It is well known that PPARγ and C/EBPα play a crucial role in adipogenesis as the cross-regulator. Therefore, our results indicate that puerarin facilitates the differentiation of adipocytes possibly by regulating the protein expression of PPARγ and C/EBPα.

### Puerarin promotes adipogenesis by activating the Akt signaling pathway

**Figure 4:**
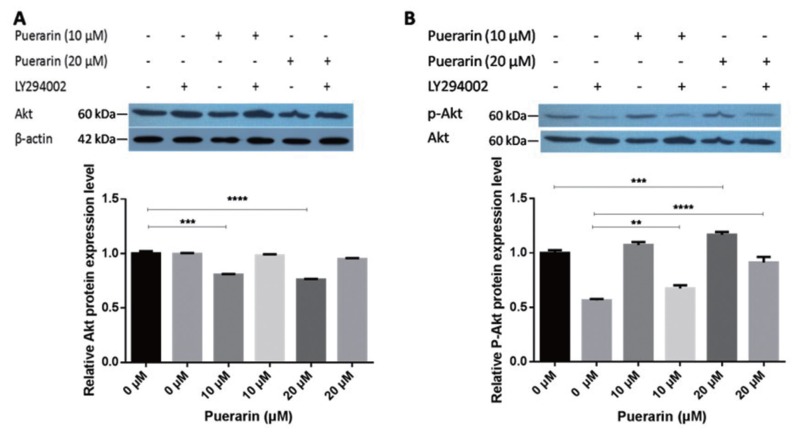
PI3K/Akt signaling is essential for puerarin regulated adipogenesis. After 8 days of differentiation in the presence of puerarin, the adipocytes were lysed, and western blotting was applied to PVDF membranes. Immunoreactive proteins were detected using a Super ECL Plus system and were quantified using Image J software. (A) The protein expression level of Akt was evaluated after incubation. The level of total β-actin was determined as a loading control; (B) The protein expression level of phospho-AKT (Ser473) was evaluated after incubation. The level of total Akt was determined as a loading control; data are presented as mean±standard deviation; ** p<0.01 compared with the control; *** p<0.001 compared with the control; **** p<0.0001 compared with the control.

In order to investigate the potential mechanism of puerarin in producing a positive effect on preadipocyte differentiation, we explored the influence of the Akt signaling pathway, a major regulator of adipocyte differentiation. Considering that Akt components are essential for the PI3K/Akt signaling pathway, we investigated the expression of Akt components and the influence of Ser473 phosphorylation during adipogenesis. As shown in Figure 4, puerarin treatment significantly decreased the protein level of Akt compared with the control group. LY294002 was added into the induced differentiation medium as a highly selective inhibitor of phosphatidylinositol 3 (PI3) kinase. The results of phosphorylation of Ser437 treated with puerarin showed that puerarin significantly facilitated the phosphorylation of Ser473 compared with that of the control group, in a does dependent manner. The phosphorylation level of Ser473 increased significantly under feedback regulation of puerarin, which was caused by LY294002. LY294002 acts as a phosphorylation enhancer to regulate adipocyte differentiation. In addition, phosphorylation levels were significantly increased by the stimulation of puerarin. These results indicated that the accumulation of lipids may be caused by promoting Ser473 phosphorylation of the Akt signaling pathway. We hypothesize that phosphorylation of Akt may be an important positive regulator of fat formation. These results indicate that puerarin promotes adipogenesis by activating Ser473 phosphorylation of the Akt signaling pathway.

## DISCUSSION

Conflicting data exists in the literature on the regulation of puerarin mediated adipogenesis and body weight gain. Wang et al [14] showed that puerarin inhibited adipogenesis and intracellular triglyceride levels, decreased the expression levels of C/EBPα, PPARγ, and fatty acid-binding protein 4. Lee, Xu, and Prasain have indicated that puerarin significantly promotes the expression of PPARγ and C/EBPα, which play a major role in the coordination of genes for adipogenic differentiation, lipid storage and maintenance of adipocyte phenotype [15–17]. We report here that puerarin induced preadipocyte differentiation which is consistent with the findings of the latter, in which an increase in the accumulation of lipid droplets and TG promoted mRNA and protein expression levels of adipogenic marker factor PPARγ. Adipocyte differentiation is regulated by adipogenic transcriptional factors such as C/EBPα and PPARγ. The main factors for difference *in vitro* may be related to the duration and concentration of puerarin supplementation. Under low concentrations of puerarin, it was shown that there is an obvious cause-effect relationship with the content of lipid droplets and triglycerides formed. However, as the concentration continues to increase, no obvious further effect on adipogenesis was found. We speculate that the possible reason is that concentration has a certain degree of influence on the growth state of the cells, which in turn produces a non-pharmacological relationship.

In the present study, the expression of PPARγ increased significantly under the addition of puerarin, while the expression of C/EBPα decreased significantly, which is consistent with the results of Rosen and Spiegelman [7]. PPARγ is the central factor of fat differentiation, and multiple CCAAT/enhancer binding morphological changes, lipid accumulation and almost all lipofuscins (C/EBP) also play a key role in adipogenesis, while C/EBPα maintains PPARγ expression later in the process. This may be the reason why CEBP levels decrease during adipogenic differentiation. PPARγ can induce the adipogenesis of fibroblasts which results in C/EBPα deficiency, while C/EBPα does not activate the fat formation switch in the absence of PPARγ. Studies have shown that PPARγ can compensate for adipogenesis disorders caused by the absence of C/EBPs and is an essential factor in the process of adipogenic differentiation [18]. However, C/EBPs cannot compensate for the effects of the *PPARγ* gene on adipogenesis [19].

The PI3K/Akt signaling pathway is a classical insulin signaling pathway [20]. Akt, known as protein kinase B or Rac, plays an important role in regulating cell growth and apoptosis. PI3 kinase affects inhibitor-sensitive pathways after activation by insulin and various extracellular signaling molecules necessary for cell growth and cell survival [21,22]. Another important function of Akt is to regulate glycogen synthesis through phosphorylation and inactivation of GSK-3α, β and regulation of insulin-stimulated glucose transport [23,24]. PI3K signaling is involved in the regulation of several cellular functions such as proliferation, differentiation, apoptosis, and glucose transport.

In order to clarify the regulatory mechanism of puerarin in adipogenesis *in vitro*, we investigated the effect of puerarin on the Akt signaling pathway. Several studies have shown that activation of the Akt pathway regulates the expression of PPARγ and C/EBPα during adipogenesis and promotes or inhibits the adipogenic differentiation of adipocytes [25–29]. In our study, puerarin dose-dependently promoted the phosphorylation of Akt Ser473, which promotes the differentiation of Yan Yellow cattle preadipocytes.

LY294002 is a highly selective inhibitor of PI3 kinase *in vitro*. LY294002 specifically inhibits PI3 kinase activity but does not inhibit other lipid and protein kinases such as PI4 kinase, PKC, MAP kinase or c-Src [30]. LY294002 has been shown to block PI3 kinase-dependent Akt phosphorylation and kinase activity. In the subsequent experiment, PI3 kinase-dependent Akt phosphorylation and kinase activity were activated by puerarin. These results show that puerarin further attenuates the phosphorylation of Ser473 caused by the inhibitor of PI3 kinase, LY294002, in the Akt signaling pathway compared with that of the control group.

In conclusion, we have shown that puerarin promotes adipogenesis of Yan Yellow cattle *in vitro*, and that the Akt signaling pathway is involved in adipogenesis. We have revealed a new perspective of puerarin in controlling treatment of lipid deposits, and this knowledge will be important for elevating beef quality.
